# Early Posterior Negativity as Facial Emotion Recognition Index in Children With Attention Deficit Hyperactivity Disorder

**DOI:** 10.32598/bcn.9.6.439

**Published:** 2018-11-01

**Authors:** Mahdiyeh Sarraf-Razavi, Mehdi Tehrani-Doost, Farnaz Ghassemi, Mohammad Ali Nazari, Zohreh Ziatabar Ahmadi

**Affiliations:** 1. Department of Neuroscience, School of Advanced Technologies in Medicine, Tehran University of Medical Sciences, Tehran, Iran.; 2. Division of Neurocognitive Sciences, Psychiatry and Behavioral Sciences Research Center, Mashhad University of Medical Sciences, Mashhad, Iran.; 3. Department of Psychiatry, School of Medicine, Tehran University of Medical Sciences, Tehran, Iran.; 4. Research Center for Cognitive and Behavioral Sciences, Tehran University of Medical Sciences, Tehran, Iran.; 5. Department of Biomedical Engineering, Amirkabir University of Technology, Tehran, Iran.; 6. Department of Psychology, Faculty of Education & Psychology, Cognitive Neuroscience Laboratory, University of Tabriz, Tabriz, Iran.; 7. Department of Speech Therapy, School of Rehabilitation, Babol University of Medical Sciences, Babol, Iran.

**Keywords:** Emotional face recognition, Event-Related Potentials (ERP), Early Posterior Negativity (EPN), Attention Deficit Hyperactivity Disorder (ADHD)

## Abstract

**Introduction:**

Studies indicate that children with Attention Deficit Hyperactivity Disorder (ADHD) have deficits in social and emotional functions. It can be hypothesized that these children have some deficits in early stages of facial emotion discrimination. Based on this hypothesis, the present study investigated neural correlates of early visual processing during emotional face recognition in this group compared with typically developing children using the Event-Related Potentials (ERPs).

**Methods:**

Nineteen boys between the ages of 7 and 11 years diagnosed with ADHD (Combined type) based on DSM-IV-TR classification were compared with 19 typically developing children matched on age and gender. The participants performed an emotional face recognition task while their brain activities were recorded using the event-related potentials procedure.

**Results:**

A significant reduction in the Early Posterior Negativity (EPN) for happy and angry faces has been revealed in ADHD children compared to normal ones (P<0.05).

**Conclusion:**

The present study supports the notion that individuals with ADHD have some impairments in early stage of emotion processing which can leading to their misinterpretation of emotion in faces.

## Highlights

- Facial expression recognition seems to be different in children with attention-deficit/hyperactivity disorder compared to normal children.
- Early posterior negativity can be utilized as an objective index to measure facial expression in children with versus without attention-deficit/hyperactivity disorder.

## Plain Language Summary

Children with Attention-Deficit/Hyperactivity Disorder (ADHD) have some impairment in emotional relationship, possibly due to deficits in emotional processing. The present study investigated neural correlates of emotional face processing in this group compared with typically developing children using the Event-Related Potentials (ERP).

A total number of 19 children diagnosed with ADHD were compared with 19 typically developing children matched with their age, gender and IQ. The participants performed an emotional face recognition while their brain activities were recorded using an ERP procedure. The pictures comprised four expressions (angry, happy, sad, and neutral). We defined four buttons for each facial expressions on a joy stick. All participants were invited to the laboratory of ERP recording. During the ERP session, the participants were seated in a comfortable chair in a dimly lit room. All children were instructed to press on the button for each expression when they recognized the target stimuli.

Current results showed reduced brain function for happy and angry recognition in ADHD children compared to normal ones. We could interpret that ADHD children have some impairments in early facial emotion processing and allocation resources of attention to emotion which can lead to their misinterpretation of emotion faces and inappropriate reaction to them in their relationship which is of special importance in understanding their cognitive-processing problems. In addition, this finding might be useful in future planning of ADHD treatments.

## 1. Introduction

Attention Deficit Hyperactivity Disorder (ADHD) is the most prevalent neurodevelopmental disorder characterized by inattentiveness and hyperactivity/impulsivity. This disorder affects 5%–12% of children and adolescents (American psychiatric association, 1994; Biederman, 2005). These children have difficulties in social and emotional functions and are often rejected by their peers (Nixon, 2001; Stormont, 2001).

Recognition of emotional expressions, as the main component of nonverbal communication, is important to regulate adaptive behaviors in social interaction (Corbett & Glidden, 2000; Williams, 2006). Studies demonstrated that children with ADHD are significantly less accurate in identifying emotional expressions compared to healthy children (Singh et al., 1998; Cadesky, Mota, & Schachar, 2000; Pelc, Kornreich, Foisy, & Dan 2006; Tehrani-Doost et al., 2017). Frontotemporal-posterior and frontostriatal-cerebellar systems and associated neuromodulators are activated in emotional functions (Durston, van Belle, & de Zeeuw, 2011). These regions are supposed to be involved in emotion deficits in ADHD (Corbett & Glidden, 2000; Dickstein, Bannon, Xavier Castellanos, & Milham, 2006; Durston et al., 2011).

Impaired recognition of emotions can be caused by problems in orienting to emotional stimuli. An eye tracking study (Ahmadi, Judi, Khorrami, Mahmoudi-Gharaei, & Tehrani-Doost, 2011) and few Event-Related Potential (ERP) studies reported that these impairments are associated with early stages of facial emotion processing and selective attention in ADHD (Williams et al., 2008; Barry et al., 2009; Nazari et al., 2010; Sarraf Razavi, Tehranidoost, Ghassemi, Purabassi, & Taymourtash, 2017), as well as autism spectrum disorders Chronaki (2016). In addition, ERP studies reported deficits in selective attention in other cognitive tasks in ADHD (Barry et al., 2009; Nazari et al., 2010). According to these results, early stage of facial emotion processing may lead to emotion recognition deficits. To evaluate this hypothesis, the neural correlates of emotion perception must be investigated in ADHD.

ERP is a research tool with high temporal resolution to investigate cognitive functions like stages of emotional processing (Calvo & Beltrán, 2014). This procedure reveals the neural correlates of cognitive processes with a precision of milliseconds (Coles & Rugg, 1995). A number of ERP components are involved in early stages of emotional processing, such as P1 (P100), N170, EPN (early posterior negativity), mainly generated from occipital or temporal regions. The other components which are mainly generated from anterior or central regions are P2, N2, VPN (vertex positive potential). N1 is another component which is widely distributed over the entire scalp (Schupp et al., 2004; Calvo & Beltrán, 2014).

P100 component is bilaterally localized in occipital areas and fusiform gyrus. It is primarily involved in visual attention and initial sensory encoding (Hillyard, Mangun, Woldorff, & Luck, 1995; Eimer & Holmes, 2002). Batty and Taylor (2003) reported increased P100 amplitude in response to negative expressions. It is controversial whether this component is modulated by emotion in face. N170 component is associated with the processing of face stimuli emerged from Superior Temporal Sulcus (STS) and Fusiform Gyrus (FG). Furthermore, the sensitivity of N170 to facial emotion is controversial (Haxby, Hoffman, & Gobbini, 2002; Itier & Taylor, 2004; Ibáñez et al., 2010). However, the sensitivity of EPN to facial emotion has been reported in several studies.

The EPN is an indicator of processing emotional valence and arousal of visual images and faces (Calvo & Beltrán, 2014). EPN reflects the degree of visual attention and early encoding of affective discrimination in both positive and negative emotions, compared to neutral ones (Schupp et al., 2004; Schacht & Sommer, 2009; Palazova, Mantwill, Sommer, & Schacht, 2011; Rellecke, Sommer, & Schacht, 2012; Calvo & Beltrán, 2014; Zhang et al., 2014). The effects of EPN on negative emotions, compared to both neutral and positive emotions (Schupp et al., 2004), as well as its positive relationship with neutral facial expressions have been reported (Holmes, Bradley, Kragh Nielsen, & Mogg, 2009).

Some studies have found larger EPNs for fear and anger compared to happiness (Schupp et al., 2004; Rellecke et al., 2012). Wieser et al. (2012) reported no EPN in patients with Parkinson disease indicating early emotion discrimination impairments. The main neural sources of this early emotion discrimination seem to be located in primary and secondary visual processing areas of the brain. The EPN is the result of a relative increase in negativity at temporo-occipital electrodes occurs between 150 and 400 ms after stimulus onset.

According to Schupp et al. (2004) EPN is sensitive to emotional stimuli, compared to neutral stimuli generated from occipito-temporal regions, between 250 and 350 ms after stimuli onset. Similarly, studies on emotional word reported a more negative-going EPN component for emotional words compared to neutral words between 200 and 300 ms and or between 300 and 400 ms (Schacht & Sommer, 2009; Palazova et al., 2011; Zang et al., 2014).

We aimed to evaluate the early stages of facial emotion processing in children with ADHD, and the EPN is a useful ERP component for this purpose. Literature reporting the neural basis of emotional dysfunction in children with ADHD is scarce. To our knowledge and according to a review article by Chronaki (2016), the current study is the first to investigate the early stages of neural activity of facial emotion recognition based on EPN component in children with ADHD. The present study aimed to better understand the early stages of facial emotion processing in children with ADHD. We hypothesized that the early emotion recognition, as reflected by the EPN, would be diminished in ADHD group for emotional faces compared with neutral ones.

## 2. Methods

### 2.1. Subjects

Nineteen boys aged between 7 and 11 (Mean±SD age: 9.21±1.13 y) years and diagnosed with ADHD were compared with 19 (Mean±SD age: 9.73±1.04 y) typically developing boys, matched on age, gender, and years of education. Typically developing children were recruited from elementary schools in Tehran, Iran. Children with ADHD (combined type) were selected from drug-naive patients referred to a child and adolescent psychiatric clinic. Diagnosis was made by a child and adolescent psychiatrist, based on DSM-IV-TR (Statistical Manual of Mental Disorders, Fourth edition, the American Psychiatric Association, 1996) criteria.

Conners’ Parent Rating Scale-Revised (short form) was administered on both groups to confirm the diagnosis of ADHD and determine severity of symptoms. The subjects with T-scores of CPRS-subscales above 65 were excluded from the normal group. Members of both groups were right-handed and had normal visual acuity. In addition, Intelligence Quotient (IQ) of all participants were evaluated according to the Wechsler Intelligence Scale for Children-Revised (WISC-R) IQ test (Mean±SD score for ADHD group: 106±4.36, for normal group: 112±10.71) (Table 1).

**Table 1:** Clinical and demographic characteristics of Typically Developing (TD) children and children with ADHD

**Characteristics**	**Mean±SD**		**t**	**P**

	**TD (n=19)**	**ADHD (n=19)**		
Age, y	9.73±1.04	9.21±1.13	0.720	0.406
Full-scale IQ	112±10.71	106±4.36	7.140	0.06
CPRS-R-oppositional	45±6.2	78±6.1	10.51	0.083
CPRS-R-inattentive	47±6.1	77±9.1	12.26	0.033^*^
CPRS-R-hyperactive	48±8.2	75±4.2	12.51	0.041^*^
CPRS-R-ADHD index	44±7.1	75±9.5	15.96	0.045^*^

CPRS: Conners’ Parent Rating Scale-Revised.

* P<0.05

### 2.2. Task and stimuli

We selected 6 faces (3 females and 3 males) in jpg format, from Cohn-Kanade AU-Coded Facial Expression Database. Each face had 4 facial expressions; happy, angry, sad, and neutral. Luminance and contrast of all images were equalized across stimuli using the Photoshop software. The photos were in black and white colors and positioned within rectangular frames (261×365 pixel array). The pictures were viewed for 2000 ms, followed by a white fixation point on the light gray background (1024×768 pixels) for 1400 ms with ±100 ms time randomization.

**Figure 1:**
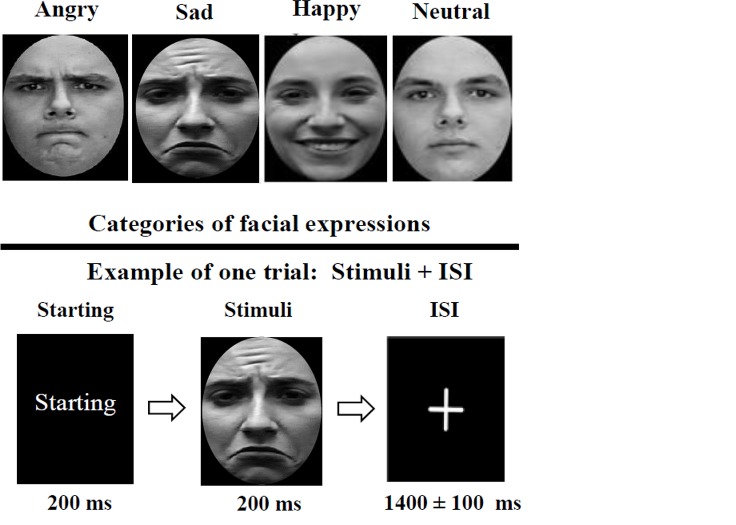
Facial expressions

The stimuli were presented on the middle of the screen. The task was designed using the Eevoke software. The task included 1 practice and 5 experimental blocks. Each block comprised 48 trials; 6 faces with 4 expressions which were repeated 2 times that began with presenting a starting picture for 200 ms. Hence, there were 60 random repeats of each expression (Figure 1). We provided 4 buttons representing each facial expression (angry, happy, sad, or neutral) on a joystick.

All participants were invited to the laboratory of ERP recording before taking any medications. A consent form was obtained from the subjects’ parents. During the ERP session, participants were seated on a comfortable chair in a dimly lit room, at 60 cm distance from a 17-inch LG computer screen. The participants were asked to always look at the center of the screen without any eye movements, if possible, and to only blink during the intervals. The subjects were monitored by a camera from another room during task performance to ensure their attendance to the stimuli. All children were instructed to press on the appropriate button once recognizing each facial expression.

Each block begins with the presentation of a starting picture for 200 ms, followed by the trial which begins with the presentation of a target stimulus (2000 ms) of the 4 stimuli of facial expressions (angry, sad, happy or neutral), followed by a fixation cross (1400±100 ms). Participants should press a key on joystick during each trial (stimuli presentation until the end of fixation). Each block comprised 48 trials.

### 2.3. Electrophysiological recording and analysis

Electroencephalography (EEG) was recorded from 64 Ag-AgCl electrodes mounted in an electrode cap (Waveguard, ANT, Netherlands) using the extended 10–20 system. ASA 4.7.1 software was used for data acquisition. Electrode impedances were maintained below 10 kΩ. The sampling rate was 512 Hz. EEG was recorded online with a 50-Hz notch filter. EEG data were analyzed offline using MATLAB R2013a software. Raw data were filtered with a band-pass filter of 0.1 to 80 Hz and referenced to the mastoids average. The eye movement artifacts were canceled using the Independent Component Analysis (ICA). In addition, the remaining artifacts with deflection amplitudes of ±100 μV were eliminated (primarily through automatic artifact reduction). Artifact-free EEG recordings were then segmented into epochs ranging from 200 ms prestimulus to 800 ms poststimulus. Each channel baseline epoch was corrected by the prestimulus voltage subtraction.

We investigated early emotion processing based on the EPN component. This component is more negative for emotional stimuli compared to neutral ones (Schupp et al., 2004). Considering the inspection of the grand average of ERPs and the literature review, we selected the mean latency of EPN component between 320 and 400 ms with a time window of 300–400 ms post-stimulus (Zhang et al., 2014) in p7, p8 electrodes.

### 2.4. Statistical analysis

Repeated measures Analysis of Variance (ANOVA) was used to analyze group differences, comprising the following core factors: hemisphere (left and right), and facial expression (happy, sad, angry and neutral) as the within-subjects variable, and the groups (patients and controls), as the between-subjects variable. Greenhouse-Geisser Correction was applied to the degrees of freedom. P<0.05 was considered as statistically significant. The Paired sample t test was used to analyze significant effects including emotional versus neutral faces. We followed-up the significant effects by a separate repeated measures ANOVA for each group (Wieser et al., 2012). Independent sample t test was used to analyze clinical and demographic characteristics of the groups.

## 3. Results

### 3.1. Analysis of demographic data

Characteristics of the ADHD and typically developing groups are summarized in Table 1. The groups were significantly different in terms of ADHD symptoms, based on CPRS-R (P<0.05).

### 3.2. Signal analysis

**Figure 2:**
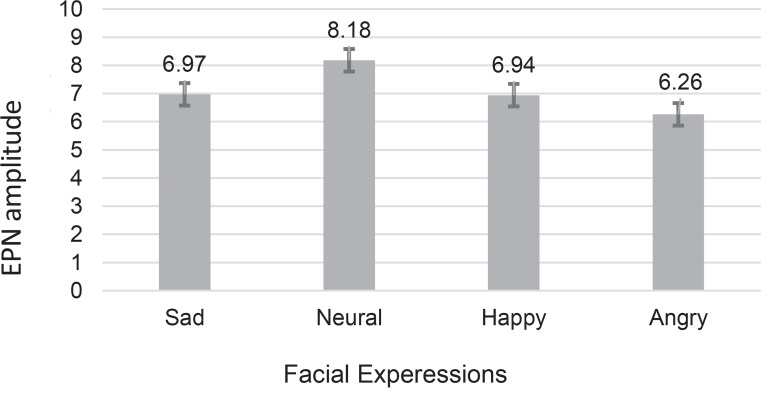
Mean scores and standard errors of EPN amplitudes for facial expression stimuli in all participants

With regard to the amplitude of EPN, there was a significant main effect of facial expression (F_2.1, 75.72_=6.25, P=0.003) using the repeated measures ANOVA. The interaction of facial expression and group was also significant in terms of EPN amplitude (F_2.1, 75.72_=8.23, P=0.001). There were no significant main effects or interaction with regard to other factors including hemisphere and facial expression, among the groups (P>0.05). Post-hoc analysis revealed significant main effects for sad (P=0.03), happy (P=0.02), and angry (P=0.001) faces, compared to the neutral ones (Figure 2). The significant interaction of facial expression with respect to the group effect indicated that such relative posterior negativity differently expressed in the groups.

**Figure 3:**
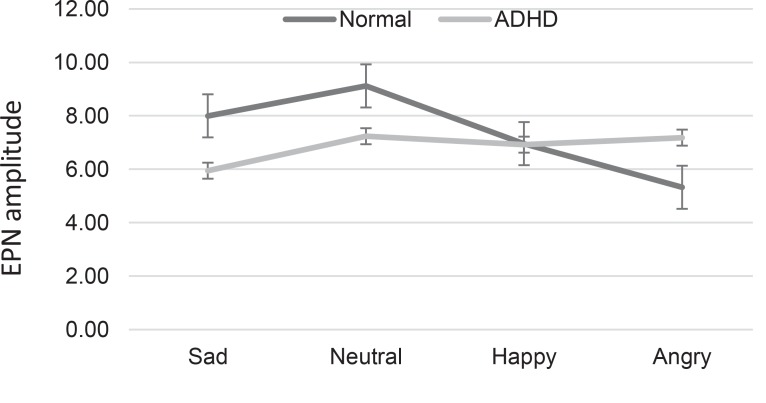
Mean scores and standard errors of EPN amplitudes for facial expression stimuli in children with ADHD and the normal group in occipito-temporal electrodes (P7/P8)

**Figure 4:**
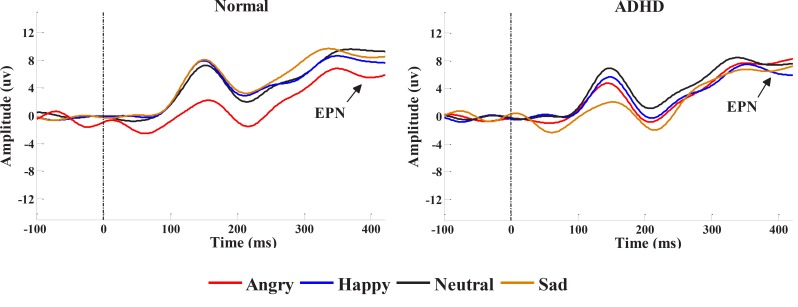
EPN amplitudes in ADHD and control groups

Repeated measures ANOVA was applied to evaluate facial emotion processing in early stages, separately for each group (Wieser et al., 2012). In normal children, a clear modulation of the EPN by facial expressions could be observed (F_1.6, 30.17_=10.87, P=0.001). Paired sample t test revealed a significant increase in the EPN amplitude for angry (P=0.00, t_18_=−7), and happy (P=0.04, t_18_=−2.2), but not sad faces (P=0.20, t_18_=−1.2), compared to neutral ones as well as angry faces compared to happy expressions (P=0.004, t_18_=3.2). There was no significant main effect of facial expression in the ADHD group (F_1.2, 21.85_=2.12, P=0.1); (sad P=0.07, t_18_=−1.9), angry (P=0.81, t_18_=−0.1), happy (P=0.3, t_18_=−1), compared to neutral faces; and for angry expressions, compared to happy faces (P=0.17, t_18_=−1.4) (Figure 3). Figure 4 illustrates the EPN amplitudes in response to neutral and emotional faces at occipito-temporal electrodes (P7/P8), separately for the children with ADHD and normal children.

## 4. Discussion

The current study aimed to evaluate the early stages of facial expression processing using the EPN component in patients with ADHD, compared to typically developing children, while viewing happy, angry, sad and neutral face images. We expected to observe emotional processing impairment in children with ADHD. The results revealed that angry and happy faces, significantly elicited the increased EPN amplitude in occipito-temporal electrodes in healthy children. However, the ADHD group showed no significant facial expression effect, based on the EPN. Thus, the obtained data supported our hypothesis that children with ADHD were different from normal children in early stages of facial expression processing.

The current results revealed a significant main effect of facial expression. Schupp et al. (2004), and Sato, Kochiyama, Yoshikawa and Matsumura (2001) reported an increased EPN in response to pictures with positive and negative facial expressions, compared to neutral faces, during 280–320 ms, and 240–300 ms poststimulus, respectively. Calvo and Beltrán (2013) reported an enlarged EPN for happy and angry faces compared to neutral, fearful, and sad expressions. According to Schupp et al. (2007), P1, N170 and EPN were related to enhanced perceptual encoding of angry expressions. In addition, happiness seems to require more detailed perceptual information that is encoded in higher visual areas and started during the EPN time window. Few studies like Kesler et al. (2001) observed the increased activation of visual processing areas by anger, compared with other facial expressions, especially in the fusiform gyrus and lateral occipital regions.

Our results revealed a modulation of the EPN component with angry and happy expressions, but not sad ones in normal children. Additionally, we observed the increased EPN measures for angry expressions, compared with happy faces in typically developing children. This finding is consistent with other studies reporting angry faces produced a larger EPN than happy faces (Balconi & Pozzoli, 2003; Rellecke et al., 2012; Calvo & Beltrán, 2014). Larger amplitudes of angry faces indicate a more general preference for threat-related faces, compared to the happy ones (Eimer & Holmes, 2007; Rellecke et al., 2012).

The facial expression had no significant effect on the ADHD group, based on the EPN component. To our knowledge, only Herrmann et al. (2009) evaluated emotional expression using the EPN component in adults with ADHD. Consistent with our results, they found significantly reduced EPN values in these individuals, compared with healthy controls, only in respect to positive stimuli. One study reported that adults with ADHD made more mistakes in recognizing happy but not angry faces (Raz & Dan, 2015). On the other hand, most behavioral studies on children with ADHD indicated impaired emotion recognition, especially for negative expressions, among them (Singh et al., 1998; Cadesky et al., 2000; Corbett & Glidden, 2000; Pelc et al., 2006).

ERP findings of William et al. (2008) are in line with our results. They reported reduced occipital P1 in adolescents with ADHD. They also reported that these individuals had difficulties in recognizing emotional expressions. They concluded that adolescents with ADHD had deficits in selective attention and early stages of emotional face processing. An eye tracking study also demonstrated that children with ADHD significantly lacked early orientation to negative facial expressions. A tendency towards negative emotions versus neutral faces was found in the normal group, while there was no such significant difference in the ADHD children (Ahmadi et al., 2011).

According to Recio, Sommer and Schacht (2011), the EPN generated bilaterally over the temporo-occipital areas implicates visual processing areas. This component represents the early emotional processing, which is an index of selective attention processes (Schupp et al., 2004). Thus, such impairments in occipito-temporal regions in children with ADHD can be interpreted as deficits in early visual pathways and selective attention or may reflect impairments in the allocation of resources to emotional face processing (Kleinhans et al., 2008; Batty, Meaux, Wittemeyer, Rogé, & Taylor, 2011). This deficit can impair emotion discrimination in the ADHD patients. However, inconsistent with our results, few studies suggest that ADHD is not associated with deficits in emotion recognition. For example, Tye et al. (2014) found no significant effect in occipito-temporal regions based on N170 amplitude in children with ADHD.

Considering our findings and previous studies, reduced EPN amplitude for emotional stimuli reveals an impairment in the early stages of emotion processing in children with ADHD. This deficit can lead to impairment in recognizing emotional faces. Based on this finding, providing attentional training to emotional stimuli may improve their impairment in recognizing emotions and lead to better emotional relationship in individuals with ADHD.

## Ethical Considerations

### Compliance with ethical guidelines

All parents filled an informed consent which was written in accordance with the Declaration of Helsinki. Children verbally agreed to participate in the experiment.
